# Spatial and Temporal Inconsistency of Forest Resilience and Forest Vegetation Greening in Southwest China Under Climate Change

**DOI:** 10.3390/plants14162493

**Published:** 2025-08-11

**Authors:** Lu Cai, Yining Luo, Yan Lan, Guoxiang Shu, Denghong Huang, Zhongfa Zhou, Lihui Yan

**Affiliations:** 1 School of Karst Science, Guizhou Normal University, Guiyang 550025, China; 232100170573@gznu.edu.cn (L.C.); luoyining@gznu.edu.cn (Y.L.); 242100170586@gznu.edu.cn (Y.L.); hdh@gznu.edu.cn (D.H.); fa6897@gznu.edu.cn (Z.Z.); 2 State Engineering Technology Institute for Karst Desertification Control, Guiyang 550025, China; 3 The State Key Laboratory Incubation Base for Karst Mountain Ecology Environment of Guizhou Province, Guiyang 550025, China; 242200091478@gznu.edu.cn; 4 School of Geography and Environmental Sciences, Guizhou Normal University, Guiyang 550025, China

**Keywords:** forest resilience, vegetation greening, BEAST, random forest, Southwest China

## Abstract

Under the backdrop of global climate warming, both forest vegetation greening and resilience decline coexist, and the consistency of these trends at the regional scale remains controversial. This study uses the kNDVI (Kernel Normalized Difference Vegetation Index) and TAC (Temporal Autocorrelation) index framework, combined with BEAST and Random Forest methods, to quantify and analyze the spatiotemporal evolution of forest resilience and its driving factors in Southwest China from 2000 to 2022. The results show the following: (1) Forest resilience exhibits a “high in the northwest and low in the southeast” spatial distribution, with a temporal pattern of “increase-decrease-increase.” The years 2010 and 2015 are key turning points. Trend shift analysis divides resilience into six types. (2) Although forest vegetation shows a clear greening trend, resilience does not necessarily increase with greening, and in some areas, an “increase in greening—decline in resilience” asynchronous pattern appears. (3) The annual average temperature, precipitation, and solar radiation are the main climate factors and their influence on resilience follows a nonlinear relationship. Higher temperatures and increased radiation may suppress resilience, while increased precipitation can enhance it. This study suggests incorporating the TAC indicator into ecological monitoring and early warning systems, along with applying trend classification results for region-specific management to improve the scientific basis and adaptability of forest governance under climate change.

## 1. Introduction

The Earth’s forest ecosystems absorb approximately 33% of anthropogenic carbon emissions, significantly mitigating the rate of global warming and playing a crucial role in the carbon cycle [1,2]. With the frequent occurrence of extreme climatic events, such as global warming, droughts and wildfires, the structure and function of forest ecosystems have been altered, the rate of their return to their original state has been disturbed, and the conversion of carbon sinks into carbon sources has reduced the carbon stock of forest ecosystems, seriously threatening the sustainable development of forest ecosystems [3,4,5]. Since Holling [6] first introduced the concept of “resilience” into the field of ecology, numerous scholars have conducted extensive research on ecosystem resilience [7,8,9]. Forest ecosystems with low resilience are more sensitive to climatic disturbances [10]. Research on the resilience of forest ecosystems can aid in the early intervention of low-resilience forest ecosystems, thereby reducing the likelihood of forest ecosystem destruction in extreme environments.

Forest resilience refers to the ability of a forest ecosystem to recover to its pre-disturbance state after external shocks, and is a key factor influencing forest functionality and stability [11,12]. A loss of resilience makes ecosystems more vulnerable and prone to tipping points due to random disturbances [13,14]. One core signal of resilience loss is the Critical Slowing Down (CSD) phenomenon, where recovery from disturbances slows significantly as the ecosystem is under prolonged pressure (e.g., climate warming or human interference) and approaches a critical threshold [15,16]. This phenomenon arises from the weakening of the system’s self-regulation capacity: as pressure accumulates, the internal negative feedback mechanisms (such as vegetation’s water retention and species complementarity) gradually fail, making it harder to counteract disturbances, leading to prolonged recovery periods [17]. Statistically, CSD is reflected in the significant increase in similarity between the system’s current state and its previous state (i.e., enhanced “memory”), and Temporal Autocorrelation (TAC) is the core indicator used to quantify this feature [18]. Specifically, TAC measures the lag-1 autocorrelation of time series (such as the correlation between vegetation indices from consecutive years), reflecting the system’s memory effect [19]. The higher the TAC value, the stronger the system’s dependence on past states, the slower the recovery, and the lower the resilience [20]. Therefore, high TAC values not only directly quantify the CSD phenomenon, but are also significantly associated with the risk of increased forest mortality, making TAC an effective early-warning indicator for assessing changes in forest resilience [14]. Currently, the TAC indicator, based on kNDVI, is widely used as an early diagnostic tool for forest resilience [21].

Global forest resilience has shown an overall decreasing trend since the beginning of the 21st century, with spatial heterogeneity [18]. Forzieri et al. explored the spatial distribution and evolutionary trends of global forest resilience from 2000 to 2020 using the TAC indicator [20]. They showed that the resilience of tropical, arid, and temperate forests is declining significantly and is associated with water resource constraints and increasing climate variability. In contrast, boreal forests have generally shown an increasing trend in resilience, possibly benefiting from warming and CO_2_ fertilization. Unlike boreal forests, the overall resilience of boreal forests is on the rise, possibly benefiting from warming and CO_2_ fertilization [18,20]. Several recent studies have explored potential factors contributing to regional heterogeneity in forest resilience. Boulton et al. suggest that climate variability may be one of several factors contributing to the loss of forest resilience in the Amazon [21]. The resilience of trees to drought is related to the water availability and the diurnal temperature range [5]. In summary, most current studies have been conducted at global or continental scales, focusing on tropical forest resilience in response to climate change, with less emphasis on regions and ecosystems outside tropical forests.

Forest ecosystem resilience is scale-dependent and influenced by spatial heterogeneity at the global scale [22]. Conclusions drawn from global-scale studies may not be applicable to regional scales and could even contradict regional realities, further emphasizing the need for region-specific studies to address local issues. Moreover, most existing global forest resilience studies focus on spatial heterogeneity and overall temporal trends in forest resilience, with less attention given to the characteristics of abrupt changes in trends over long time scales, as well as to the fine-grained classification of forest resilience trends.

Remote sensing data play a crucial role in monitoring forest ecosystems, particularly in assessing vegetation health, cover dynamics, and resilience [23]. The MODIS (Moderate Resolution Imaging Spectroradiometer) has been widely utilized in large-scale forest resilience studies due to its long-term data continuity and global coverage [14]. However, with advances in satellite technology, the use of Sentinel-1, Sentinel-2, and Sentinel-3 satellites in enhancing vegetation monitoring and forest resilience research has increased [19,24]. These platforms offer several advantages, such as higher spatial resolution, additional spectral bands, and an increased temporal frequency, which are critical for monitoring fine-scale forest structures and disturbances [25]. While the Sentinel platforms provide more detailed spatial and spectral information, the MODIS was selected in this study due to its long-term continuity from 2000 onward, which is essential for capturing broad-scale forest resilience trends over an extended period.

Long-term satellite data show that, since the 1980s, there has been a significant greening trend in global forested areas, driven by factors such as human land-use management, climate change, and CO_2_ fertilization [26,27]. However, recent studies have revealed that climate change has reduced vegetation resilience globally, particularly in highly valuable tropical forests [18,20,22,28]. The positive impacts of vegetation greening on forest resilience may be offset by background climate change and increased climate variability induced by climate change [20,22]. In the context of global climate change, the simultaneous greening of vegetation and loss of forest resilience increases uncertainty regarding the future of forest ecosystems [29]. Wang et al. explored the relationship between vegetation greenness and vegetation resilience on the Loess Plateau from 2000 to 2020 [30]. They found that vegetation on the Plateau exhibited an overall greening trend, while vegetation resilience showed an increasing trend followed by a decrease, with 2010 serving as the turning point. Furthermore, the greening of vegetation did not necessarily lead to an increase in resilience. Exploring the empirical relationships between changes in forest resilience, the dynamics of forest vegetation, and environmental factors is crucial for a comprehensive understanding of the processes shaping forest ecosystems and for the development of effective management strategies.

Southwest China is predominantly covered by subtropical vegetation, with abundant forest resources and an exceptional carbon sequestration capacity, making it the largest terrestrial carbon sink in China over the past 30 years [31,32,33]. The frequent occurrence of droughts and other extreme climatic events in recent years has significantly threatened the health of forest vegetation, compounded by ecological and environmental issues such as soil erosion, land degradation, and karst desertification, leading to an increasingly fragile regional ecosystem [34,35]. In addition to climate factors, forest resilience is also influenced by vegetation indices, topography, and human activities [19,36]. Indices like the NDVI and EVI reflect vegetation health and productivity, while topographical features affect the recovery process by regulating microclimates and the water supply [37,38]. Human disturbances such as land-use changes, population pressure, and ecological projects (e.g., afforestation) can reshape the forest structure and function [20]. As an ecologically fragile region heavily impacted by both climate and human disturbances, Southwest China provides a unique window for studying the dynamics of forest resilience [37]. These factors interact in the region’s complex mountainous environment, collectively driving the spatiotemporal evolution of forest resilience. Although ecological projects such as soil erosion control and returning farmland to forests have significantly improved the ecological quality and vegetation cover [39] and forest vegetation has shown a clear greening trend, the stability of the forest ecosystem remains uncertain [40,41]. Some studies have focused on the resilience of vegetation in the region, but most have concentrated on overall trend changes and responses during drought years [37,42]. There is still a lack of in-depth exploration into the detailed classification of forest resilience trends and their coupling mechanism with the greening of forest vegetation.

However, existing studies still lack a systematic characterization of the evolution mechanisms of forest resilience in Southwest China, as well as a quantitative identification of the driving factors. Therefore, this study aims to address the following research questions: (1) What are the spatiotemporal trends of forest resilience in Southwest China over the past two decades? (2) How are these trends related to forest greening patterns? (3) What are the key driving factors behind changes in forest resilience? This study focuses on Southwest China, using the time series of the kernel-normalized difference vegetation index (kNDVI) and the time-varying Temporal Autocorrelation (TAC) with lag-1 as the CSD indicator, combined with the BEAST model, to analyze and classify the spatiotemporal trends of forest resilience from 2000 to 2022. Additionally, we employ the Random Forest–Shapley Additive Explanations (SHAP) framework to assess the relative importance of the key drivers influencing forest resilience, providing a comprehensive perspective on the stability of forest ecosystems in Southwest China.

## 2. Results

### 2.1. Spatial and Temporal Changes in Forest Resilience

**Figure 1:**
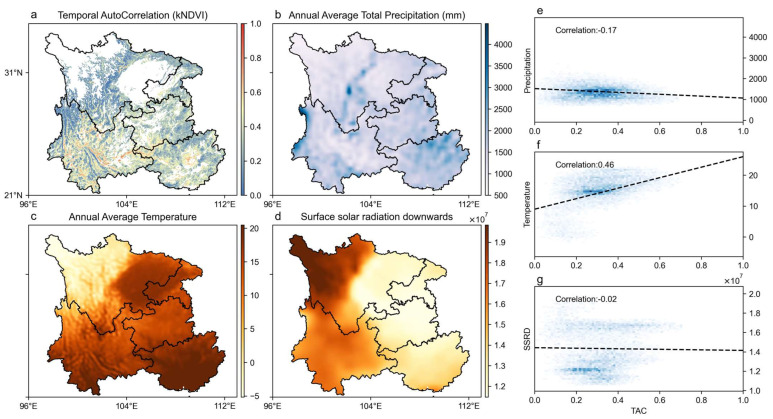
Spatial distribution of forest resilience and climatic factors (2000–2022). (**a**) Spatial distribution map of TAC. (**b**) Spatial distribution map of mean annual precipitation. (**c**) Spatial distribution map of annual mean 2 m air temperature. (**d**) Spatial distribution map of annual mean surface solar downward radiation. (**e**–**g**) Pearson correlation maps between TAC and the corresponding climatic variables, computed on a per-pixel basis using 23 years (2000–2022) of annual mean data.

The spatial and temporal distribution of forest resilience in Southwest China from 2000 to 2022 is shown in Figure 1. The larger the TAC value, the lower the forest resilience; conversely, the smaller the TAC value, the higher the forest resilience. Overall, forest resilience in the southwest region shows a distribution pattern of high in the north and low in the south, with the highest forest resilience in the northwest gradually decreasing along the southeast direction. Forest resilience in the southwest is relatively low. On the one hand, the western region is part of the Hengduan Mountains and high-altitude areas. These regions have high vegetation cover, but their ecosystems are relatively fragile and less stable [43,44,45]. On the other hand, extreme droughts and wildfires frequently occur in western regions such as Sichuan, Yunnan, and Guizhou, where external disturbances can cause forests to exhibit exceptionally low resilience [37,42].

The annual average precipitation in the southwest region is concentrated in 900–1600 mm, of which the average precipitation in central Sichuan and northeastern Guangxi is larger; the annual average temperature is the lowest in northwestern Sichuan and gradually increases along the southeast direction; and the annual average downward solar radiation maximum occurs in northwestern Sichuan and the value of the radiation decreases gradually to the east. Correlation analysis showed that TAC was positively correlated with the mean annual temperature, indicating that forest resilience tends to decline as the temperature increases. A weak negative correlation was found between TAC and annual precipitation (R = −0.17), but a clear non-linear relationship emerged. Specifically, TAC slightly increased when the precipitation was below approximately 2000 mm, whereas it declined significantly when precipitation exceeded this threshold, suggesting a possible precipitation-driven threshold effect. In contrast, the correlation between TAC and surface downward solar radiation was very weak (R = −0.02). The linear trend indicated a minimal association; however, a slight decline in TAC was observed under extremely high radiation levels, implying a potential non-linear response under extreme conditions.

**Figure 2:**
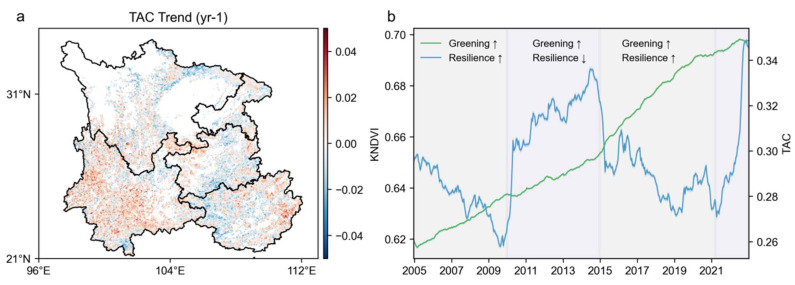
Spatial patterns and temporal trends of TAC from 2000 to 2022. (**a**) Spatial plot of TAC time trends. Positive TAC values indicate a decrease in the rate of recovery and thus a decrease in resilience. (**b**) Temporal trajectory of forest resilience and vegetation dynamics, 2000–2022.

The time trend of TAC from 2000 to 2022 was obtained using a five-year sliding window processing method (Figure 2). It can be observed that the TAC series in the southwest region generally follows a decreasing–rising–receding pattern, while the temporal change in forest resilience displays a “strengthening-weakening-strengthening” trend, with significant turning points in 2010 and 2015. Correspondingly, the average kNDVI value, also calculated over a five-year sliding window, increased continuously from 0.62 to 0.69 over the period 2000–2022, indicating a continuous greening trend of the forest vegetation as a whole from 2000 to 2022. Comparative analyses of the overall trend show that the greening of forest vegetation and the enhancement of forest resilience are not fully consistent, exhibiting “synchronous-asynchronous-synchronous” changes over the study period. This result is consistent with the findings of Wang et al. [46].

### 2.2. Classification and Spatial Distribution Characteristics of Forest Resilience Trends

**Figure 3:**
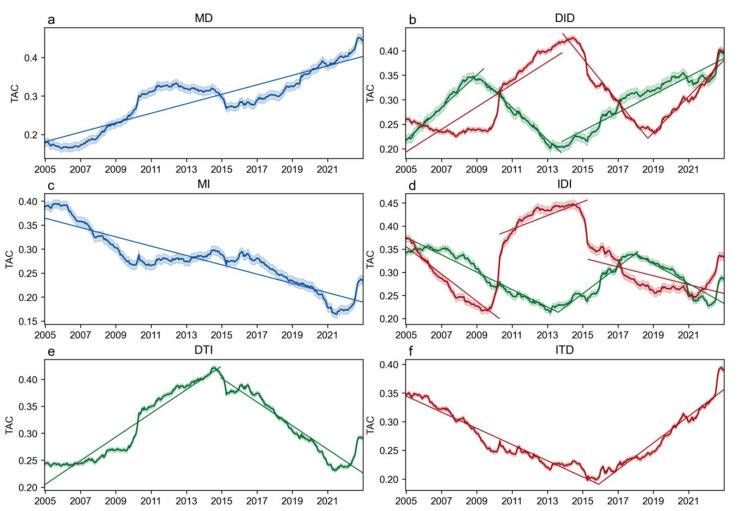
Trend variation of forest resilience (TAC) time series after classification. The six trend types include (**a**) Monotonically Decreasing (MD), (**b**) and Decreasing–Increasing–Decreasing (DID), (**c**) Monotonically Increasing (MI), (**d**) Increasing–Decreasing–Increasing (IDI), (**e**) Decreasing then Increasing (DTI), (**f**) Increasing then Decreasing (ITD). Among them, the IDI and DID types each include two typical curves: Type I is represented by green and Type II by red, distinguishing internal breakpoint timing differences. However, since their overall trends are consistent, they are analyzed as a single type.

**Figure 4:**
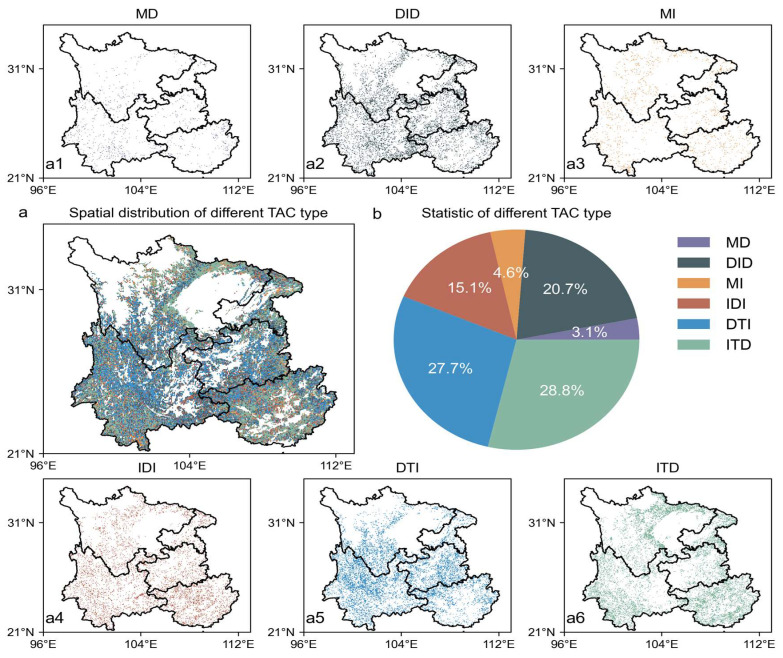
Spatial distribution and proportion of six forest resilience trend types (TAC). (**a**) Overall spatial distribution of the six forest resilience trend types. (**b**) Proportional distribution map of the six forest resilience trend types.

After performing a trend classification analysis of the TAC time series using the BEAST model, we found that over 90% of the forest resilience pixels in Southwest China experienced trend changes during the study period (Figure 3 and Figure 4). Based on the number of breakpoints and the trend changes before and after them, all the pixels in the region were classified into six trend types: the “Monotonically Decreasing” (MD) type, which accounts for 3.1% of the pixels in Southwest China, and the “Monotonically Increasing” (MI) type, which accounts for 4.6%. Both of these types did not show any breakpoints in the time series, indicating that forest resilience in these regions changed monotonically without any trend reversal. The “Increasing then Decreasing” (ITD) type and the “Decreasing then Increasing” (DTI) type account for 28.8% and 27.7%, respectively, each with one breakpoint, representing a single trend change in forest resilience. The “Increasing then Decreasing then Increasing” (IDI) type and the “Decreasing then Increasing then Decreasing” (DID) type account for 15.1% and 20.7%, respectively, each with two breakpoints, reflecting a multi-stage fluctuation process in forest resilience. It is important to note that there are differences in the timing of the turning points within the IDI and DID types. The typical subtypes (Type I and Type II) are represented by green and red curves, respectively, in the figure. Since their overall trends are consistent, they are treated as a single type in the statistical analysis. Overall, except for the MD and MI types, the remaining types account for 92.4%, indicating that most regions in Southwest China experienced at least one trend change during the study period, highlighting the dynamic and staged nature of the forest recovery process.

In terms of spatial distribution patterns, the forest resilience trend types in Southwest China exhibit significant regional heterogeneity (Figure 3 and Figure 4). The most prevalent trend type, “Increasing then Decreasing” (ITD, 28.8%), is mainly distributed in the peripheral areas of Southwest China, including the southwestern part of Yunnan and northern Guangxi. Despite ongoing vegetation greening in this region, forest resilience began to decline after reaching its peak around 2013–2015, indicating a weakening of ecosystem resilience and a potential risk of degradation. The second most common trend type, “Decreasing then Increasing” (DTI, 27.7%), is primarily located in the central areas of Southwest China, such as northern Yunnan, southern Sichuan, and western Guizhou. Since around 2015, resilience has been continuously improving, reflecting a trend of systematic recovery. The least common type, “Monotonically Decreasing” (MD, 3.1%), is concentrated in central and western Yunnan, showing a continuous degradation state, which warrants particular attention. Time series analysis also reveals that the period of 2015–2016 marked an important turning point for multiple trend types, coinciding with key time points of climate drivers (e.g., fluctuations in precipitation and temperature) identified in previous studies. This further highlights the staged nature of the regional ecosystem recovery process and its potential relationship with external pressures.

**Figure 5:**
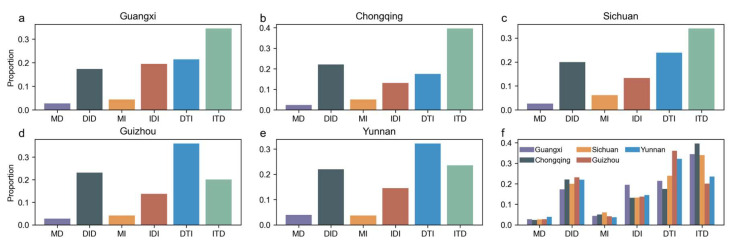
Proportional distribution of six forest resilience trend types (TAC) in Southwest China. (**a**–**e**) show the proportions of the six forest resilience trend types in each of the five provinces (Guangxi, Chongqing, Sichuan, Guizhou, and Yunnan). (**f**) provides a comprehensive comparison of these proportions across the five provinces to highlight regional differences.

The overall pattern also shows certain regularities at the provincial scale, as shown in Figure 5. The proportions and structures of the different trend types vary across the provinces. The ITD and DTI types dominate in Yunnan, Guizhou, Sichuan, Chongqing, and Guangxi, indicating that the forest resilience trends in Southwest China are primarily characterized by non-monotonic patterns, including “Increasing then Decreasing” and “Decreasing then Increasing.” Guangxi shows a prominent proportion of the ITD type, while Chongqing and Guizhou are characterized by the DTI type. The MD type in Guizhou and Yunnan has a relatively higher proportion, reflecting ongoing local ecological degradation. In contrast, the distribution of trend types in Sichuan and Chongqing is more balanced, suggesting a relatively stable state of the forest ecosystem. Figure 5f further compares the proportions of forest resilience trend types across the southwestern provinces, highlighting regional differences. For instance, Guangxi’s ITD type is significantly dominant, while Yunnan’s MD type stands out, reflecting variations in the response and recovery modes of forest ecosystems to external disturbances.

To investigate forest resilience (TAC) responses to climate factors, multiple linear regression analyzed relationships between TAC and precipitation (TP), temperature (T2M), and solar radiation (SSRD) across trend types (Table 1). The goodness of fit varied significantly: monotonic types (MD, MI) showed higher R^2^ (0.8123, 0.8459), indicating primary climatic control, while reversal types (IDI, DID, DTI) had moderate R^2^ (0.5735–0.6872), suggesting additional influences. Precipitation had the strongest association, with positive correlations in specific types (e.g., DID-I: 0.0203, IDI-I: 0.0614) and continuous nonlinear effects, though no sharp threshold was detected. Temperature and SSRD generally showed weak negative correlations (e.g., T2M: −0.0439, SSRD: −0.0393), implying stress from warming and high radiation.

In summary, precipitation is the primary climatic driver of resilience variations, with extreme levels linked to significant changes despite no distinct threshold. Its continuous nonlinear influence, supported by regression coefficients, outweighs the weaker negative impacts of temperature and radiation. These findings highlight regional resilience heterogeneity and provide a mechanistic basis for trend classifications, emphasizing precipitation’s key role.

**Figure 6:**
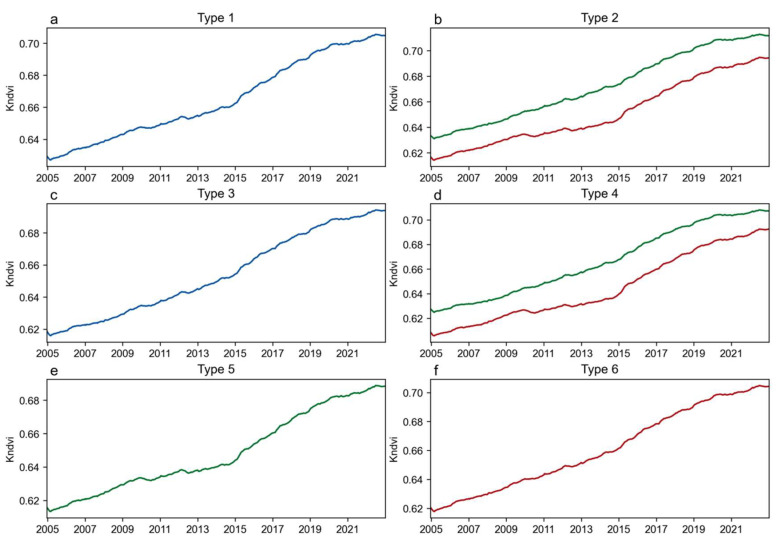
Time-series trend plots of the mean kNDVI values of the corresponding image elements for different TAC trend types. (**a**–**f**): Type 1–Type 6 correspond to the time series trend of kNDVI for the same image elements of MD, DID, MI, IDI, DTI, and ITD, respectively. Among them, the IDI and DID types each include two typical curves, with Type I represented in green and Type II in red. Since their TAC trend types are consistent, they are analyzed as a single type.

The same sliding window method used for TAC was applied to calculate the mean kNDVI time series, resulting in a time series trend plot that shows the mean kNDVI values corresponding to the forest resilience classification (Figure 6). The results indicate that the mean kNDVI values of the image elements corresponding to the TAC trend classifications exhibit a continuous increasing trend, and the forest resilience under different trend classifications displays the same inconsistency with the greening of forest vegetation.

### 2.3. Coupling of Forest Vegetation Greening and Forest Resilience

In the context of overall forest vegetation greening in Southwest China, different trend types of forest resilience exhibited distinct response mechanisms. The kNDVI and the temporal correlation between the TAC time series of different trend types reflected the spatial correspondence between the two trends, further validating the observation that forest vegetation dynamics and forest resilience in Southwest China did not change in unison during the study period.

**Figure 7:**
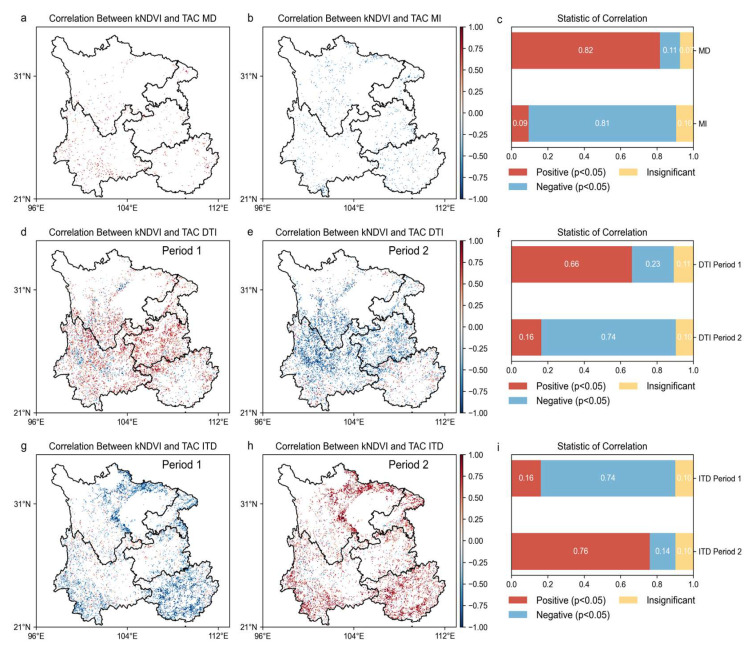
Correlation of forest vegetation dynamics (kNDVI time series) with forest resilience (TAC time series). (**a**) Spatial correlation maps for MD; (**b**) spatial correlation maps for MI; (**d**,**e**) spatial correlation maps for different stages of DTI; (**g**,**h**) spatial correlation maps for different stages of ITD; (**c**,**f**,**i**) coverage scores for each correlation type.

**Figure 8:**
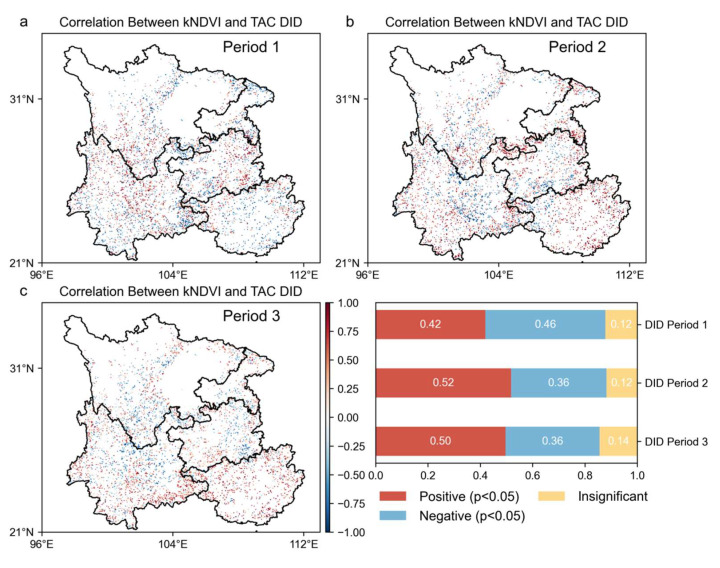
Correlation plot between vegetation dynamics (kNDVI time series) and forest resilience (TAC time series) at different stages of DID. (**a**) Spatial correlation map of DID type in the first declining stage; (**b**) spatial correlation map of DID type in the increasing stage; (**c**) spatial correlation map of DID type in the re-declining stage.

**Figure 9:**
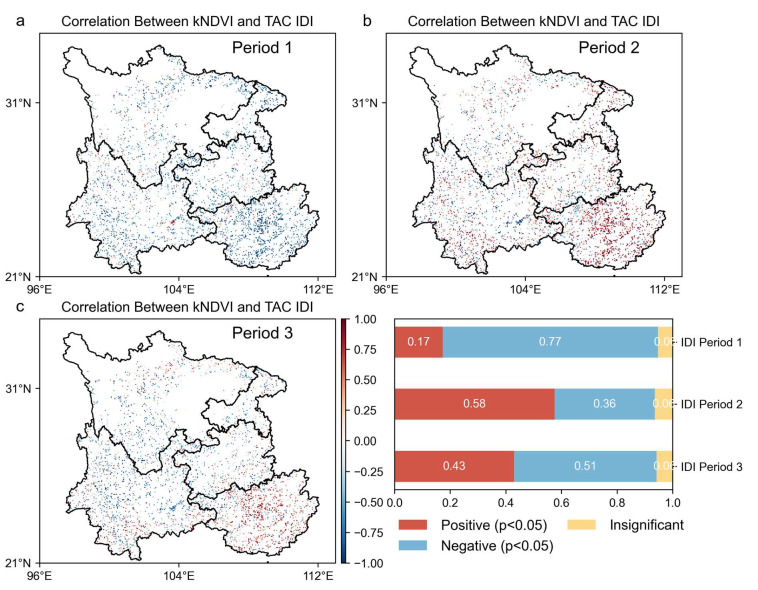
Correlation plot between vegetation dynamics (kNDVI time series) and forest resilience (TAC time series) at different stages of IDI. (**a**) Spatial correlation map of IDI type in the first increasing stage; (**b**) spatial correlation map of IDI type in the declining stage; (**c**) spatial correlation map of IDI type in the re-increasing stage.

The image elements with a significant negative correlation (*p* < 0.05) between the kNDVI and TAC indicate that forest resilience increases while forest vegetation grows. The results are shown in Figure 7, Figure 8 and Figure 9. In the stage of increasing resilience, 81% of the pixels in MI showed an increase in forest resilience along with forest vegetation growth. Similarly, forest resilience was synchronized with the greening of forest vegetation in DTI’s Period II, Type 6’s Period I, ITD’s Period II, and IDI’s Periods I and III. The percentage of image pixels in each stage was 74%, 74%, 36%, 77%, and 51%, respectively. The results show that forest resilience is increasing with the greening of forest vegetation in the southwest. During the resilience decline stage, 82% of the pixels in MD showed a decrease in forest resilience while forest vegetation was growing. Similarly, the inconsistency between forest resilience and forest vegetation greening also occurred in DTI’s Stage I, ITD’s Stage II, DID’s Stages I and III, and IDI’s Stage II. The percentage of pixels in each stage was 66%, 76%, 42%, 50%, and 58%, respectively. The results show that there is an inconsistency between forest vegetation dynamics and changes in forest resilience. Forest resilience does not increase with the greening of forest vegetation.

High kNDVI and low TAC trends do not always correspond, suggesting that the greening of forest vegetation does not necessarily lead to increased forest resilience over long time scales. Overall, there is an inconsistent pattern of change between forest resilience and forest greening or browning. These results highlight that, although forest cover greening is expected to increase productivity and biomass, it does not necessarily result in a sustained increase in forest resilience.

### 2.4. Analysis of Drivers of Forest Resilience

**Figure 10:**
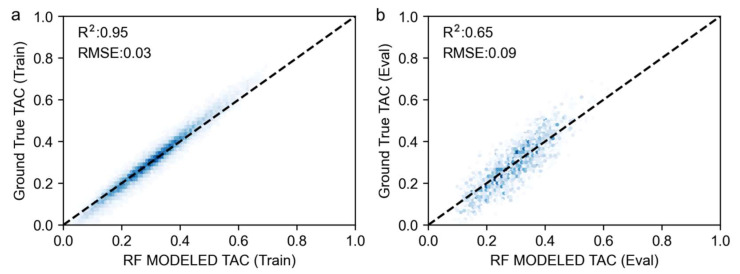
Scatter Plots of Random Forest-Predicted TAC Versus Ground-Truth TAC. (**a**) Random Forest-Predicted TAC vs. Ground-Truth TAC in the Training Set; (**b**) Random Forest-Predicted TAC vs. Ground-Truth TAC in the Evaluation Set.

The relative importance of factors affecting forest resilience change in Southwest China was quantitatively analyzed using the RF model in combination with the SHAP explanatory framework. The results of the RF analysis showed that the forest resilience change was significantly affected by local environmental conditions, as shown in Figure 10 (R^2^ = 0.66, RMSE = 0.09).

**Figure 11:**
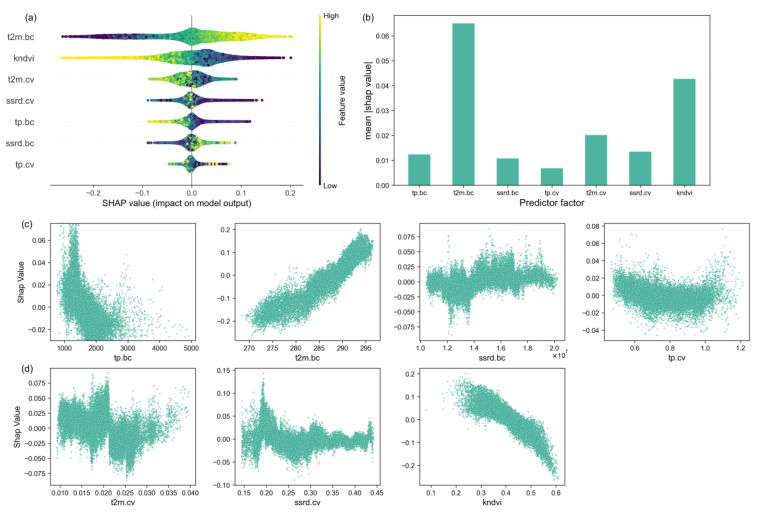
Ranking of importance of potential drivers to TAC change values based on the RF-SHAP analysis framework. (**a**) The overall importance of each variable. The y-axis shows the importance ranking of influencing factors and the x-axis shows the average SHAP values of each factor. (**b**) The overall importance and direction of influence of variables. Yellow (blue) dots indicate high (low) values of influencing factors. SHAP > 0 means promoting TAC changes (i.e., suppressing forest resilience), and SHAP < 0 means inhibiting TAC changes (i.e., enhancing forest resilience). (**c**,**d**) SHAP dependence plots: the relationship between TAC and each climate driver.

The SHAP correlation plot illustrates the relationships between SHAP values for each feature. Negative SHAP values indicate a reduction in TAC from the baseline value caused by specific variables. Based on the variables’ overall importance ranking, the annual mean temperature (t2m.bc) and kNDVI are the primary drivers of TAC change. When SHAP values decline, kNDVI increases, thereby bolstering forest resilience (Figure 11).

Among the climate drivers, changes in the mean annual temperature and radiation conditions were the main factors contributing to the shift from enhancement to a loss of forest resilience. An increase in the mean annual temperature (t2m.bc) shows a positive contribution to TAC, leading to a decrease in forest resilience, while the mean annual precipitation (tp.bc) played the opposite role. And the effect of the temperature on forest resilience was greater than that of precipitation, and the average state of both had a greater effect on forest resilience than the variability, which is consistent with the results of previous studies. An increase in the mean annual downward surface solar radiation (ssrd.bc) hinders the increase in forest resilience. The effects of climate variability on forest resilience all showed quadratic relationships, and it can be observed that temperature variability (t2m.cv), precipitation variability (tp.cv), and surface downward solar radiation variability (ssrd.bc) affect forest resilience in a non-linear manner. When temperature variability was small or large, SHAP values increased, weakening forest resilience. However, when temperature variability was moderate at about 0.025, the forest vegetation showed progressively weaker autocorrelation (indicating increased forest resilience). Overall, in southwestern China, lower temperatures, reduced solar radiation, and increased precipitation are more likely to show increased forest resilience.

## 3. Discussion

### 3.1. Types of Spatial and Temporal Distribution Patterns and Trends in Forest Resilience

From 2000 to 2022, forest resilience in southwestern China exhibited a “strengthening–weakening–strengthening” trend, with critical turning points in 2010 and 2015. These shifts in forest resilience were linked to regional climate changes, vegetation succession, and human activities [47]. Jiang et al. demonstrated that the drought-affected areas in Southwest China from 2009 to 2011 were consistent with the spatial distribution of low forest resilience, and that the severe drought event in 2010 played a significant role in the transition from increasing to decreasing forest resilience [37]. Meanwhile, the implementation of ecological protection and restoration projects by the State, such as the Pearl River Defense Project, the Yangtze River Defense Project, the Return of Cultivated Land to Forest, and the Comprehensive Management of Rocky Desertification in southwestern China [48,49], has significantly increased vegetation cover and enhanced greening in typical areas like western Guizhou and northern Yunnan, strongly contributing to the rebound of forest resilience [50].

More than 90 percent of forest resilience in Southwest China experienced a shift in trend, with the highest percentage of ITD types at 28.7 per cent and the lowest percentage of MD types at 3.1 percent. Recent studies have shown that a temporary loss of resilience can serve as an early warning signal for predicting forest mortality [51]. Although forest resilience of the persistently declining type (MD), mainly in the Three Rivers region of west–central Yunnan Province, is the smallest proportion, the risk of forests in this region facing persistently declining resilience should not be ignored. Low resilience means that ecosystems have a lower capacity to resist environmental disturbances and recover more slowly after disturbances, increasing the risk of transition to alternative regimes [22]. Therefore, areas of forest resilience loss need special attention and protection to lay the foundation for future ecological restoration.

### 3.2. Coupling Forest Vegetation Greening with Forest Resilience

Forest vegetation greening does not necessarily correspond to enhanced forest resilience [30]. Although greening is generally associated with increased vegetation cover, higher biodiversity, and an improved community structure—all of which theoretically support ecosystem stability—recent studies suggest that this relationship is not always straightforward [18,20]. In Southwest China, despite extensive forest greening over the past two decades, trend analysis reveals a decline in forest resilience in several regions [52]. This decoupling between greening and resilience aligns with global findings, which indicate that while forest carbon sequestration and primary productivity may increase, ecosystem vulnerability can also rise [20,29,53]. Furthermore, the presence of green vegetation does not necessarily indicate overall ecosystem health, as the functioning of ecosystems is often constrained by the environmental carrying capacity [54,55].

These discrepancies may arise due to multiple ecological constraints, particularly in karst regions where shallow soils, a limited water availability, and climatic extremes inhibit sustainable vegetation growth [56]. Additionally, the biogeophysical effects of greening and increasing climate variability may offset its expected ecological benefits [20,22]. Our results reveal a “synchronous–asynchronous–synchronous” temporal pattern between vegetation greening and resilience trends in Southwest China. Among the six trend types identified by the BEAST model, distinct asynchrony was observed. For example, in the DID and IDI trend types, 42% and 58% of the pixels, respectively, exhibited inconsistent trends between kNDVI and TAC, suggesting that greening does not always predict resilience improvements. This discrepancy is especially pronounced in ecologically fragile karst regions, where a complex terrain, limited resources, and high climatic variability further exacerbate the issue [56].

This trend of asynchrony carries significant implications for forest management and early warning systems. Regions where vegetation appears to be greening but resilience is declining may harbor hidden degradation risks [30]. Identifying such areas enables proactive monitoring and targeted interventions. For instance, incorporating TAC as an early warning indicator within forest management frameworks could help detect critical transitions before ecosystems cross irreversible thresholds. Furthermore, the six trend types provide a refined classification of ecosystem trajectories, offering valuable insights for implementing differentiated management strategies across heterogeneous landscapes. In conclusion, while forest greening reflects certain aspects of ecological productivity, it should not be equated with ecosystem health or stability. Effective forest monitoring and policy frameworks should integrate resilience indicators to ensure robust, adaptive, and sustainable management under the pressures of climate change and land-use change.

### 3.3. Drivers of Change in Forest Resilience

Climate change plays a key role in driving fluctuations in ecosystem resilience. In Southwest China, changes in the mean annual temperature and radiative conditions are the primary factors contributing to the transition from enhanced to declining forest resilience. Among these, temperature exerts a stronger influence on forest resilience than precipitation. Moreover, the mean state of both temperature and precipitation has a greater impact on forest resilience than their interannual variability [22].

In Southwest China, lower temperatures, reduced solar radiation, and increased precipitation are more likely to be associated with enhanced forest resilience. In contrast, rising temperatures have a significant inhibitory effect on forest resilience. The temperature can enhance regional ecosystem productivity by extending the growing season, promoting photosynthesis, and regulating nutrient cycling [57,58]. However, the potential threats that elevated temperatures pose to ecosystem resilience should not be overlooked. Even with increased precipitation, the accompanying rise in soil water evapotranspiration can offset the benefits of soil moisture replenishment and may even lead to an increase in the number of drought days [59]. This is particularly critical in Southwest China, where karst landscapes are widespread [60]. The shallow soil layers and highly developed fissures in these regions result in a low natural water retention capacity, making vegetation more vulnerable to water stress. Such stress can significantly inhibit both vegetation growth and its capacity to recover. In addition, increased surface solar radiation has been shown to hinder forest resilience. Forzieri et al. emphasized that forest resilience declines significantly once a critical threshold of solar radiation is exceeded. Elevated radiation intensifies evapotranspiration, thereby exacerbating water stress and adversely affecting plant growth and ecosystem resilience [20]. The effect of solar radiation on forest resilience exhibits a non-linear response pattern, highlighting the complexity of its underlying mechanisms and underscoring the importance of understanding vegetation responses to environmental change. Moreover, the significant roles of precipitation and kNDVI in enhancing forest resilience further indicate that resilience is the result of synergistic interactions among multiple environmental factors. The inter-relationships among these variables warrant further investigation in future studies.

### 3.4. Shortcomings and Outlook

This study reveals the spatiotemporal dynamics of forest resilience (TAC) in Southwest China from 2000 to 2022, identifying several regions with sustained resilience loss. Despite an overall greening trend across the region, our findings demonstrate that vegetation greening does not necessarily indicate enhanced ecosystem resilience. Furthermore, we find that changes in climatic factors—particularly warming and growing water constraints—are the primary drivers of resilience shifts. These findings align with previous studies showing that continued warming and intensified drought have slowed vegetation recovery rates in terrestrial ecosystems [21,36,61]. However, this study focuses solely on retrospective trend analysis and lacks projections under future climate scenarios. Future research should integrate multi-scenario climate simulations to forecast resilience trajectories, thereby providing an early-warning basis for ecological risk management under changing environmental conditions.

Regarding data sources, this study employed MODIS NDVI products due to their long-term temporal coverage, broad spatial extent, and public availability. MODIS data have been widely applied in resilience assessments at both regional and global scales, particularly for detecting early-warning signals and threshold behavior in vegetation systems under climatic stress [18,20,21,22,61,62]. Although the spatial resolution of MODIS is lower than that of newer high-resolution datasets (e.g., Sentinel-2), it remains well-suited for tracking large-scale patterns and long-term dynamics [24]. Nonetheless, future studies could integrate high-resolution remote sensing data to better characterize local ecological processes and improve responsiveness to spatial heterogeneity and fine-scale disturbances

At the methodological level, TAC, which is derived from the theory of critical slowing down (CSD), has gained increasing recognition as a proxy for forest resilience and an early-warning indicator, as supported by recent studies [20,36,63]. However, it is crucial to understand that CSD-based indicators are primarily designed to detect signals of declining system resilience and should not be viewed as precise predictors of imminent regime shifts. To enhance the policy relevance and practical applicability of TAC, we suggest two key directions for future research: (1) integrating additional ecological indicators (e.g., community composition and vegetation cover dynamics) and utilizing multi-source remote sensing data to improve the sensitivity and spatial applicability of TAC; and (2) further exploring the relationship between CSD signals and actual ecological transitions, thereby fostering the development of resilience metrics as actionable tools for ecosystem management and informed decision making.

## 4. Materials and Methods

### 4.1. Study Area

**Figure 12:**
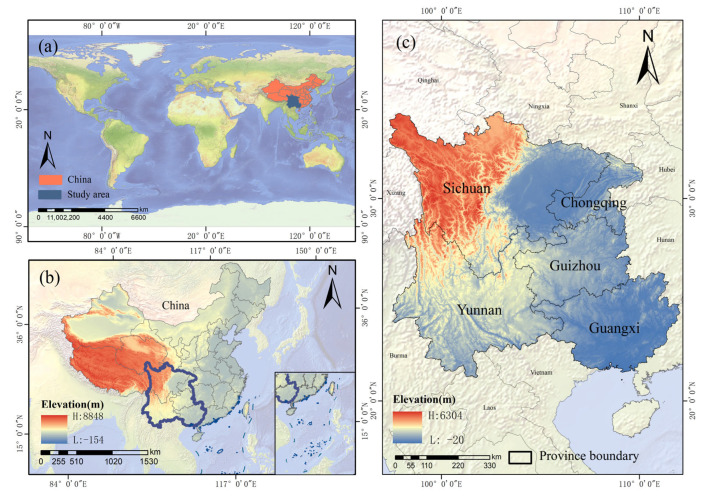
Location of the study area. (**a**) Location of the study area in the world; (**b**) Elevation map of the study area in China; (**c**) Elevation map of the study area.

The study area is located in Southwestern China (20° N–35° N, 97° E–113° E), encompassing five provinces (municipalities and autonomous regions): Chongqing, Sichuan, Guizhou, Yunnan, and Guangxi, covering a total area of 1.37 million km^2^ (Figure 12). The terrain of the region is characterized by higher elevations in the northwest and lower elevations in the southeast, with a complex and varied topography that forms a multifaceted geomorphological system, including basins, plateaus, mountains, and coasts. The climate is predominantly subtropical monsoon, with annual precipitation exceeding 1000 mm [34]. The region’s abundant water and heat resources, combined with significant vertical climatic differentiation, have created a comprehensive vegetation spectrum, ranging from monsoon rainforests to alpine meadows. As a global biodiversity hotspot and the largest terrestrial carbon sink in China over the past 30 years, the region is rich in forest resources and has a substantial carbon sequestration capacity [32]. For many years, the implementation of ecological projects, such as soil erosion prevention and the conversion of farmland to forests, has significantly improved vegetation cover and ecological quality. However, frequent climatic disasters and widespread karst landscapes have increased the sensitivity of forest ecosystems to disturbances, becoming a key constraint on ecosystem stability in the region [31].

### 4.2. Data Sources

Remotely sensed vegetation data were used to calculate forest resilience, while auxiliary datasets were utilized for the attribution analyses of forest resilience. The dataset includes MOD13C1 data, land use type classification data, and ERA5 reanalysis data. Detailed information about the dataset is provided in Table 2.

All the aforementioned data were resampled to a spatial resolution of 0.05° using the nearest neighbor method, and data processing was conducted in the MATLAB environment (version R2023a). To extract forested areas in Southwest China, the land cover product was used. The annual mean and variability of the meteorological variables were calculated based on the temperature, precipitation, and surface downward solar radiation data from the ERA5 reanalysis dataset. These variables included the annual mean temperature (t2m.bc), annual mean precipitation (tp.bc), annual mean surface downward solar radiation (ssrd.bc), temperature variability (t2m.cv), surface precipitation variability (tp.cv), and downward shortwave radiation variability (ssrd.cv). These variables were used to analyze the climatic drivers of forest resilience.

kNDVI is a novel vegetation index that can indirectly and accurately measure the dynamics of terrestrial carbon sources and sinks, effectively addressing the issue of vegetation saturation in densely vegetated areas [64]. It can serve as a key indicator for describing the state of forest ecosystems and has been validated in numerous studies [20,30,45]. kNDVI is derived from the NDVI, as shown in Equation (1).

(1) $$kNDVI={tanh(NDVI)}^{2}$$

### 4.3. Methods

#### 4.3.1. Forest Resilience Indicators

Ecosystem resilience can be quantified by the rate at which a system returns to a steady state after disturbance [7]. Under natural conditions, forest ecosystems exhibit significant seasonal fluctuations due to the cyclical processes of vegetation growth, development, and decline [12,34]. High-intensity disturbances, such as droughts and extreme temperatures, can disrupt the system’s homeostasis, resulting in a gradual reduction in the rate of return to equilibrium, a phenomenon known as critical slowdown (CSD) [4,6]. CSD is characterized by a high degree of autocorrelation in state variables, whereby the system’s state tends to remain similar across consecutive observations. An increase in lag-1 autocorrelation serves as an intuitive indicator of critical slowdown. The temporal autocorrelation function (TAC) is used as a proxy for forest resilience [20,63], quantifying the degree of autocorrelation to characterize the ecosystem’s resistance to disturbance and recovery dynamics.

Before calculating the TAC values, the time series need to be smoothed. First, the kNDVI data were de-seasonalized and de-trended to generate the kNDVI anomaly series. The TAC was then calculated based on the anomalous component of the kNDVI time series. The overall TAC time series from 2000 to 2022 was calculated to obtain the spatial distribution of forest resilience, which was used to analyze the impact of climatic factors. Additionally, the long-term resilience trend may mask short-term fluctuations in resilience [62]. To better understand the temporal trends of forest resilience in Southwest China, the TAC time series was calculated at the pixel level using a sliding window of 5 years (60 months). A positive TAC value indicates an increase in resilience over time, while a negative value indicates a decrease in forest resilience. The equation for forest resilience TAC is expressed in Equation (2):

(2) $$Z_{t+1}=TAC*Z_{t}+\varepsilon_{t}$$

where $Z_{t}$ is a subset of the ecological state series (i.e., kNDVI time series anomalies), $Z_{t+1}$ is a first-order lag time series, TAC is the autoregressive coefficient, and $\varepsilon_{t}$ is the residual obtained by ordinary least squares.

#### 4.3.2. Forest Resilience Classification Methodology

The Bayesian Estimator of Abrupt Change, Seasonality, and Trend (BEAST) is a Bayesian model–averaged time-series analysis framework that effectively captures nonlinear and abrupt features in time series, allowing for a fine-grained classification of forest resilience trends [65]. Extensive testing on synthetic data and various remote sensing time series products has demonstrated that the BEAST can accurately capture true nonlinear dynamics within time series [66,67,68].

The BEAST decomposes the periodic time series data into trend, seasonal, and residual components. The seasonal and trend signals may contain multiple mutation points. The BEAST detects these mutation points and divides the time series into several sub-series. A harmonic model is used to fit the seasonal signal and a linear model is used to fit the trend signal, thereby splitting the overall trend into multiple distinct sub-trends. The method can be implemented in MATLAB and the related program package can be downloaded from https://github.com/zhaokg/Rbeast. The TAC time series with a sliding window of five years was input into the BEAST for detection, and forest resilience was classified into six trend types based on the number of mutation points and the direction of the trend slope of the subsequences. The results are shown in Table 3.

#### 4.3.3. Methodology for Analyzing Drivers of Forest Resilience

(1) Random Forest Regression Model

Random Forest (RF) is a non-parametric machine learning model that excels at capturing complex non-linear relationships [69,70]. RF can uncover interactions and non-linear dependencies between predictor variables, making it more effective than traditional linear models for analyzing ecosystem dynamics. It is particularly well-suited for integrating the complex effects of various factors on forest resilience (TAC) [20].

To explore the drivers of forest resilience (TAC) using the Random Forest (RF) model, vegetation characteristics (kNDVI) and climate variables were selected as potential drivers and input into the model. The dataset was randomly divided into two subsets: a training set containing 70% of the data for model calibration and a test set with the remaining 30% for model validation. The model performance was evaluated using two metrics: the coefficient of determination (R^2^) and the root mean square error (RMSE) [71]. A higher R^2^ indicates a better model fit, while a lower RMSE suggests smaller estimation errors. The evaluation metrics were calculated as follows:

(3) $$R^{2}=1-\frac{\sum_{i=1}^{n}{\left(y_{i}-{\hat{y}}_{i}\right)}^{2}}{\sum_{i=1}^{n}{\left(y_{i}-\bar{y}\right)}^{2}}$$

(4) $$RMSE=\sqrt{\frac{\sum_{i=1}^{n}{\left({\hat{y}}_{i}-y_{i}\right)}^{2}}{n}}$$

where $y_{i}$ represents the true value of TAC, ${\hat{y}}_{i}$ represents the value predicted by the TAC model, $\bar{y}$ is the mean value of the measured TAC, and *n* represents the sample size.

(2) Shapley Additive Explanations

Shapley Additive Explanations (SHAP) are a machine learning model interpretation framework based on Shapley value theory from cooperative game theory [72]. It provides both global and local interpretability by decomposing prediction results to assess the impact of each feature [73]. The SHAP framework directly quantifies the marginal contribution of each feature (e.g., precipitation) to the model’s outcome, thus monitoring its impact on the dependent variable (SHAP value) [74]. A higher SHAP value indicates a greater marginal contribution of a feature to the TAC value. For a sample *x* with M features, the sum of the explained attribute values for each feature *i* shall be the output of the model *f*(*x*), which is expressed by the formula shown in Equation (5) as:

(5) $$f\left(x\right)=\varphi_{0}+\sum_{i=1}^{M}\varphi_{i}X_{i}$$

where *f*(*x*) is the original predicted value of sample *x*, is the expected value of the predicted value, and is the SHAP value of feature *i*.

## 5. Conclusions

In this study, we developed a forest resilience assessment framework based on the temporal autocorrelation (TAC) of the kernel normalized difference vegetation index (kNDVI) time series. We also applied the BEAST model to systematically analyze the evolution of forest resilience trends and their driving mechanisms in Southwest China from 2000 to 2022. The main conclusions are as follows:

(1) Forest resilience in Southwest China exhibited a spatial pattern of “high in the northwest and low in the southeast,” with a temporal evolution characterized by a “strengthening–weakening–strengthening” trajectory. Key turning points were identified around 2010 and 2015.
(2) More than 90% of forest pixels experienced at least one trend shift during the study period, which could be categorized into six typical trajectories. Among them, the “increase-to-decrease” (ITD, 28.8%) and “decrease-to-increase” (DTI, 27.7%) types were dominant, while the “monotonically decreasing” (MD, 3.1%) type was mainly concentrated in central–western Yunnan, representing areas at high risk of ecological degradation. These dynamic patterns highlight the nonlinear and stage-dependent nature of forest resilience.
(3) Vegetation greening does not necessarily indicate improved resilience. During the resilience enhancement phase, 81% of pixels in the MI (Monotonic Increase) category exhibited synchronous increases in both the kNDVI and resilience. However, in the resilience degradation phase, 82% of MD-type pixels showed an asynchronous pattern, where “greening occurred while resilience declined.” Similar mismatches were observed across other trend types, suggesting that the kNDVI alone cannot reliably reflect the true recovery status of ecosystems. The risk of “greening masking degradation” should not be overlooked.
(4) Forest resilience was primarily driven by climatic factors, exhibiting nonlinear and threshold responses. SHAP analysis identified key variables such as the mean annual temperature, precipitation, and kNDVI. Warming and increased solar radiation were found to weaken resilience, while increased precipitation contributed to resilience enhancement. The effects of climatic variability on resilience followed a quadratic relationship, highlighting the sensitivity of ecosystems to climate disturbances in different contexts.

In summary, we recommend incorporating the TAC indicator into forest monitoring and early warning systems. Coupled with trend classification results, this approach will facilitate regionally differentiated management and intervention, thereby improving the scientific and adaptive capacity of forest governance in the context of climate change.

## Figures and Tables

**Table 1:** Multiple linear regression results of TAC against precipitation (TP), temperature (T2M), and surface solar radiation (SSRD) across different trend types.

TAC Type	Intercept	Precipitation (TP)	Temperature (T2M)	Surface Solar Radiation (SSRD)	R^2^
MD	0.2918	–0.0440	0.0415	–0.0393	0.8123
DID-Ⅰ	0.2889	0.0203	0.0417	0.0279	0.5763
DID-Ⅱ	0.3056	–0.0710	–0.0075	–0.0453	0.6872
MI	0.2775	0.0279	–0.0439	0.0258	0.8459
IDI-Ⅰ	0.2882	0.0614	–0.0127	–0.0404	0.5735
IDI-Ⅱ	0.3229	–0.0634	–0.0340	–0.0431	0.5778
DTI	0.3142	–0.0573	–0.0018	–0.0192	0.5932
ITD	0.2704	0.0309	–0.0027	0.0099	0.2332

**Table 2:** Spatial and temporal resolutions and sources of datasets.

Product	Spatial Resolution	Temporal Resolution	Source
MOD13C1 MODIS NDVI	5 km	16-day	https://earthdata.nasa.gov/
GLC SHARE	1 km	Yearly	https://www.un-spider.org/
Temperature	0.1°	monthly	ERA5 https://www.un-spider.org/
Precipitation	0.1°	monthly	
Surface Solar Radiation Downward	0.1°	monthly	

**Table 3:** The different types of forest resilience change detected by BEAST.

Type Name	Meanings
Monotonic increase (MI)	No obvious mutation was detected and the overall trend showed a monotonic increase.
Monotonic decrease(MD)	No obvious mutation was detected and the overall trend showed a monotonic decrease.
Increase then decrease (ITD)	One obvious mutation was detected and the trend shifted from an increase to a decrease.
Decrease then Increase (DTI)	One obvious mutation was detected and the trend shifted from a decrease to an increase.
Increase–decrease–increase (IDI)	Two obvious mutations were detected and the trend shifted from an increase to a decrease, then reversed back to an increase.
Decrease–increase–decrease (DID)	Two obvious mutations were detected and the trend shifted from a decrease to an increase and then to a decrease again.

## Data Availability

MOD13C1 NDVI data can be obtained from NASA Earth data (https://lpdaac.usgs.gov/products/mod13c1v006/), accessed on 1 January 2025. ERA5 Land were obtained from the Google Earth Engine platform: https://earthengine.google.com (accessed on 1 January 2025). GLC SHARE data can be obtained from https://www.un-spider.org/ (accessed on 1 January 2025).
